# Gas sensors boosted by two-dimensional h-BN enabled transfer on thin substrate foils: towards wearable and portable applications

**DOI:** 10.1038/s41598-017-15065-6

**Published:** 2017-11-09

**Authors:** Taha Ayari, Chris Bishop, Matthew B. Jordan, Suresh Sundaram, Xin Li, Saiful Alam, Youssef ElGmili, Gilles Patriarche, Paul L. Voss, Jean Paul Salvestrini, Abdallah Ougazzaden

**Affiliations:** 1 CNRS, UMI 2958, GT - CNRS, 2 rue Marconi, 57070 Metz, France; 2 Georgia Institute of Technology, School of Electrical and Computer Engineering, GT-Lorraine 57070 Metz, France; 3 Institut Lafayette, 57070 Metz, France; 4 GT Lorraine, UMI 2958, GT - CNRS, 2 rue Marconi, 57070 Metz, France; 5 Centre de Nanosciences et de Nanotechnologies, CNRS, Univ. Paris-Sud, Université Paris-Saclay, C2N – Marcoussis, 91460 Marcoussis, France

## Abstract

The transfer of GaN based gas sensors to foreign substrates provides a pathway to enhance sensor performance, lower the cost and extend the applications to wearable, mobile or disposable systems. The main keys to unlocking this pathway is to grow and fabricate the sensors on large h-BN surface and to transfer them to the flexible substrate without any degradation of the performances. In this work, we develop a new generation of AlGaN/GaN gas sensors with boosted performances on a low cost flexible substrate. We fabricate 2-inch wafer scale AlGaN/GaN gas sensors on sacrificial two-dimensional (2D) nano-layered h-BN without any delamination or cracks and subsequently transfer sensors to an acrylic surface on metallic foil. This technique results in a modification of relevant device properties, leading to a doubling of the sensitivity to NO_2_ gas and a response time that is more than 6 times faster than before transfer. This new approach for GaN-based sensor design opens new avenues for sensor improvement via transfer to more suitable substrates, and is promising for next-generation wearable and portable opto-electronic devices.

## Introduction

AlGaN/GaN-based sensors with catalytically active gate electrodes are an interesting sensing technology due to their many advantageous material properties, such as high thermal and chemical stability^1^, which have demonstrated sensitivity and suitability for detection of diesel exhaust gases^2,3^. With appropriate transfer technique, AlGaN/GaN sensors have promising potential to be integrated into wearable applications. This would enable the development of a wearable and portable air pollutant monitoring platform to collect air pollutant data (species and concentration) continuously. Such a system requires inexpensive, miniaturized (micro-scale), rapidly responding and highly sensitive gas sensors that can be operated on malleable and lightweight substrates. Existing Metal Organic Vapour Phase Epitaxy (MOVPE) for GaN-based sensors uses fabrication on a rigid substrate, typically sapphire or silicon, and epi-layers cannot be easily released from the substrate because of the strong bonding. Several options for the release of epi-layers exist including fielded laser lift-off^4^ for GaN devices and chemical etching of the growth substrate^5^ or a sacrificial layer^6^. These techniques have many limitations in practice, notably high cost, long process times, and limitations in size.

Van der Waals epitaxy, which involves the growth of sp^3^ bonded semiconductor layers on 2D materials^7^, allows active layer release from its substrate and transfer to a foreign support^8,9^. This promises to reduce manufacturing cost by permitting re-use of the native substrate. It can also enable device integration into low-cost substrate foils, which can be used for foldable, wrapped, rollable and portable systems^10^. Hexagonal boron nitride (h-BN) is a III-N material which exhibits a two-dimensional structure when grown as monolayers of nanometer thicknesses, similar to graphene. It is particularly compatible with growth of wurtzite III-N devices in a single growth run. AlGaN/GaN HEMT and InGaN-based LEDs have been grown and fabricated on h-BN^11,12^, but large-scale device fabrication has not been yet demonstrated.

In this work, we report wafer-scale fabrication of AlGaN/GaN gas sensors grown on 2-inch sapphire wafers with a sacrificial 2D layered h-BN by MOVPE. In addition to transferring sensor devices to a flexible substrate, we demonstrate an enhancement in gas sensing performance of these devices. This is in part due to the effect of foreign substrate choice on the temperature in the two-dimensional gas (2DEG) channel of the AlGaN/GaN heterostructure, which is validated by thermal modeling and Raman spectroscopy. We also analyze the strain changes in the semiconductor materials after release from the sapphire layer, and consider its effect on device performance. The sensors are tested before and after transfer to the flexible substrate with significant improvements in both sensitivity and response time after the transfer. This approach for engineering of GaN-based sensors is a key step in the pathway towards economically viable, flexible sensors with improved performances that could be integrated into wearable applications.

**Figure 1:**
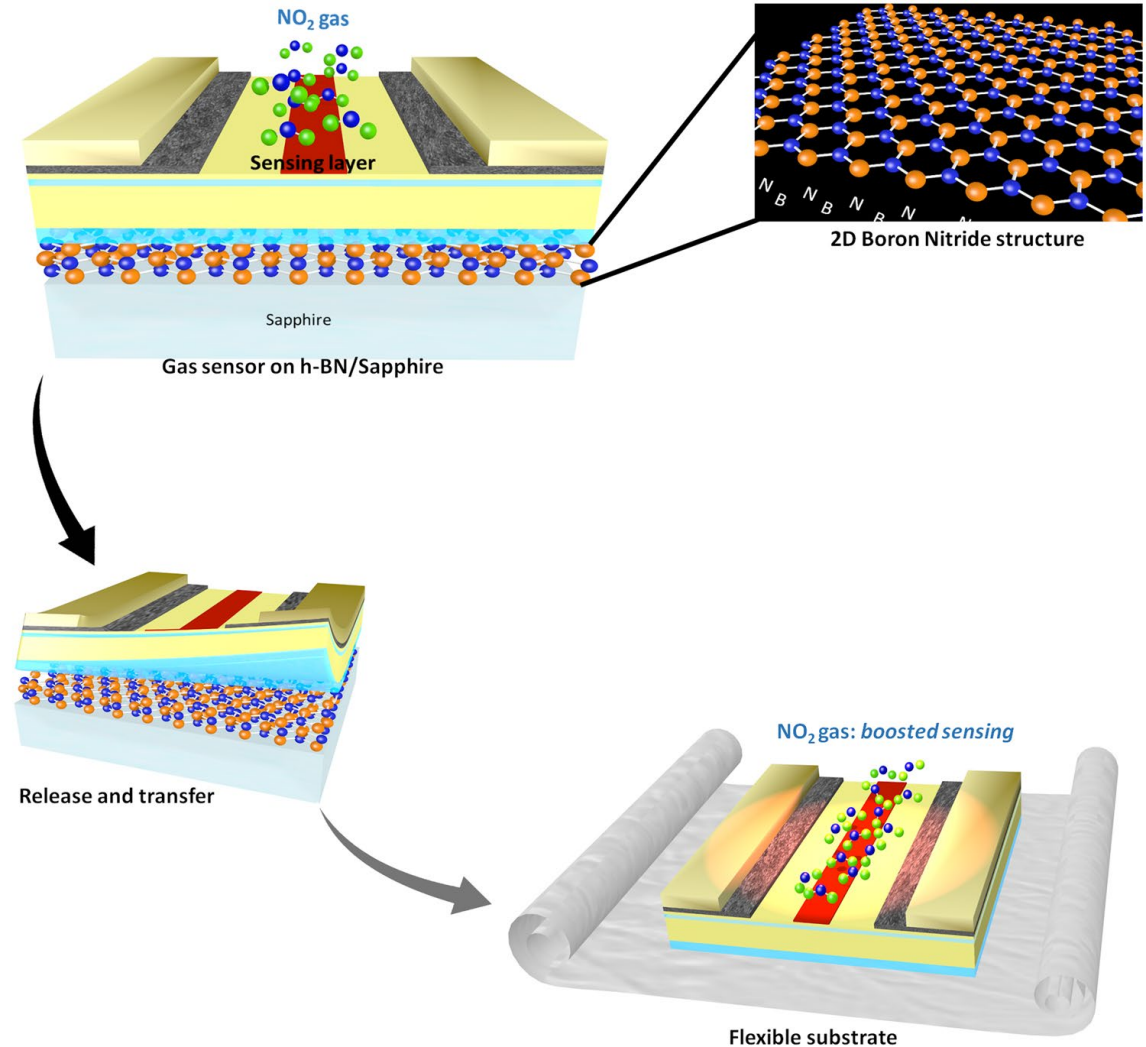
Our approach for the growth, fabrication, release and transfer (see Method section for details) of boosted AlGaN/GaN gas sensor to a flexible sheet using h-BN as a buffer and release layer.

The mechanism for boosted gas sensor operating on a foreign substrate like a flexible carrier is illustrated in Fig. 1. The 2D h-BN (the crystallographic phase of the grown h-BN has been demonstrated in a previous study^13^) layer enables the release of the device and its transfer to an acrylic adhesive layer. This layer is expected to confine the heat generated by self-heating, leading to higher operating temperature of the gas sensor, which is known to contribute to an increase of the sensitivity and an improvement of the response time^2^.

### Structural characterization of AlGaN/GaN structure on sapphire

**Figure 2:**
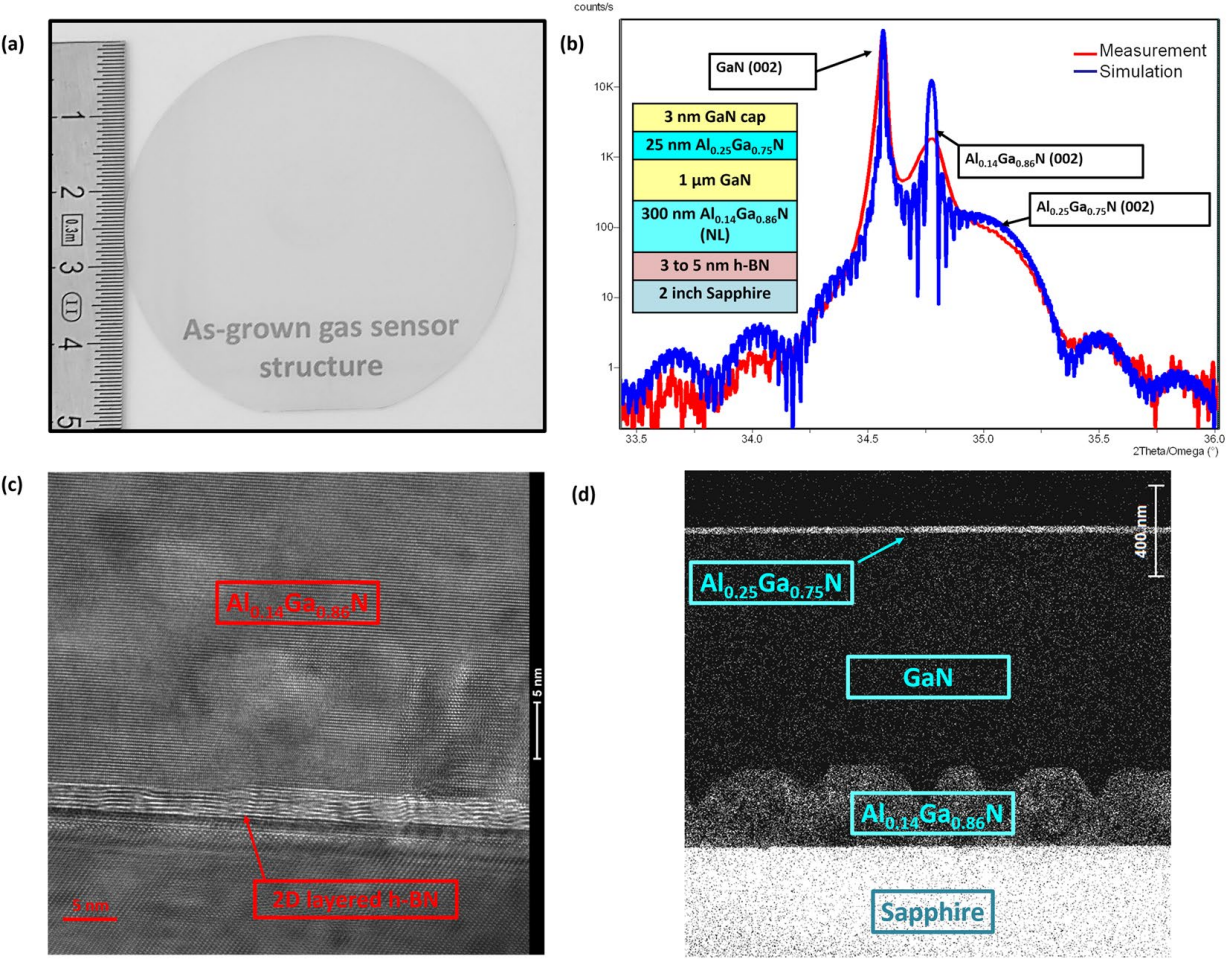
(**a**) Photo of the grown AlGaN/GaN on h-BN, (**b**) High resolution X-ray diffraction 2θ − ω scans of the grown AlGaN/GaN heterostructure on h-BN using Al_0.14_Ga_0.86_N as a nucleation layer (NL), the red curve is the measurement and the blue curve presents the simulation result. The inset is a schematic of the grown structure. (**c**) High resolution TEM image showing the interface between the 2D layered h-BN and the AlGaN nucleation layer. (**d**) Energy dispersive X-ray spectroscopy (EDX) elemental mapping of Al.

The AlGaN/GaN structure was grown by MOVPE on 2-inch h-BN/sapphire substrates (Fig. 2a). The high-resolution X-ray diffraction (HRXRD) 2θ − ω scan of the grown layers on h-BN/sapphire is presented in Fig. 2b. The satellite peaks from the Al_0.25_Ga_0.75_N/GaN heterostructure were clearly observed, along with GaN (002) and Al_0.14_Ga_0.86_N (002) diffraction planes. Simulation, shown in blue, confirms the Al content and the thicknesses of the different layers in the structure. The broadness and the low intensity of the AlGaN buffer compared to the simulated one are due to the 3D morphology of this layer as shown in the elemental EDX mapping of Al in Fig. 2d. The high resolution TEM image in Fig. 2c is a direct evidence of the crystal phase and quality of the 5 nm thick h-BN layer. An abrupt interface without inter-diffusion along the Al_0.25_GaN_0.75_/GaN can be seen in energy dispersive X-ray spectroscopy (EDX) element mapping in Fig. 2d.

### Electrical characterization of AlGaN/GaN devices on sapphire

**Figure 3:**
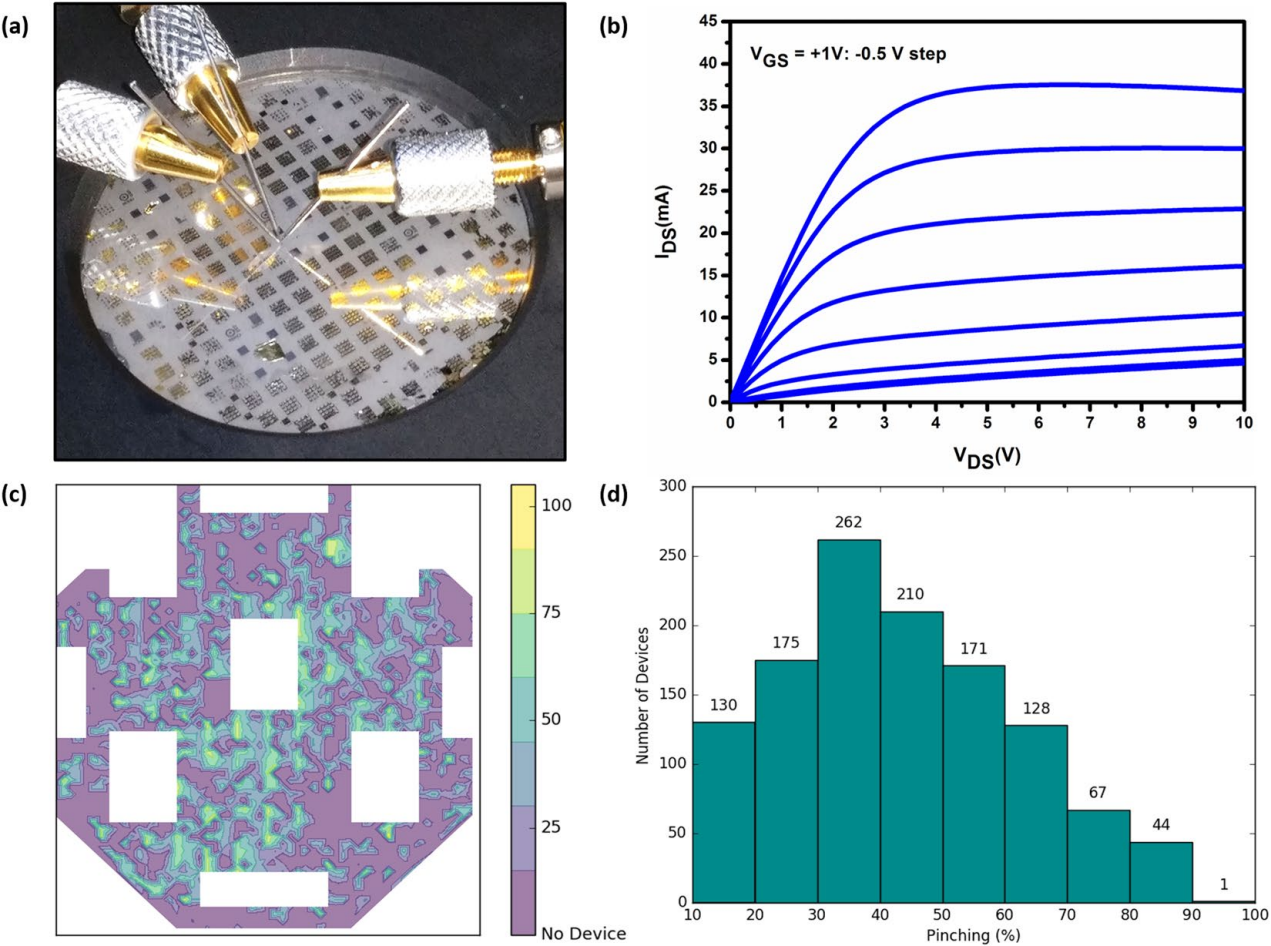
(**a**) Photo of the wafer-scale processed AlGaN/GaN sensors, (**b**) I_DS_-V_DS_ characteristic of an AlGaN/GaN device with 2 µm gate length (**c**) Wafer mapping of the gate pinching, with white areas representing masked-off regions that include TLM patterns (**d**) Histogram of the pinching distribution across the wafer.

The process fabrication has been adapted for device structures on 2D h-BN. For instance, no ultrasonic cleaning is used, rapid thermal annealing conditions such as gas flow rate and temperature ramping were optimized, and contact with liquids was limited during each step to prevent spontaneous delamination^14^. The resulting processed device is shown in Fig. 3a. Transfer length measurements (TLM) performed on several locations of the wafer show an average specific contact resistivity of 3 × 10^−5^ Ω.cm^−2^, indicating good ohmic contact behavior. Wafer mapping of the current-voltage characteristics reveal more than 1000 functional AlGaN/GaN devices on the 2-inch wafer (Fig. 3b,c); processing optimizations can further improve the yield. We also note that more than 100 devices exhibit more than 70% gate pinching, which we have quantified as [I_ds_(V_gs_ = 0 V) − I_ds_(V_gs_ = −6V)**/**I_ds_(V_gs_ = 0 V)]*100 at V_ds_ = 10 V. This is a direct indication that these devices have good control of the carrier concentration in the 2DEG by the gate contact (Fig. 3d).

### Sensor measurements: before and after transfer

**Figure 4:**
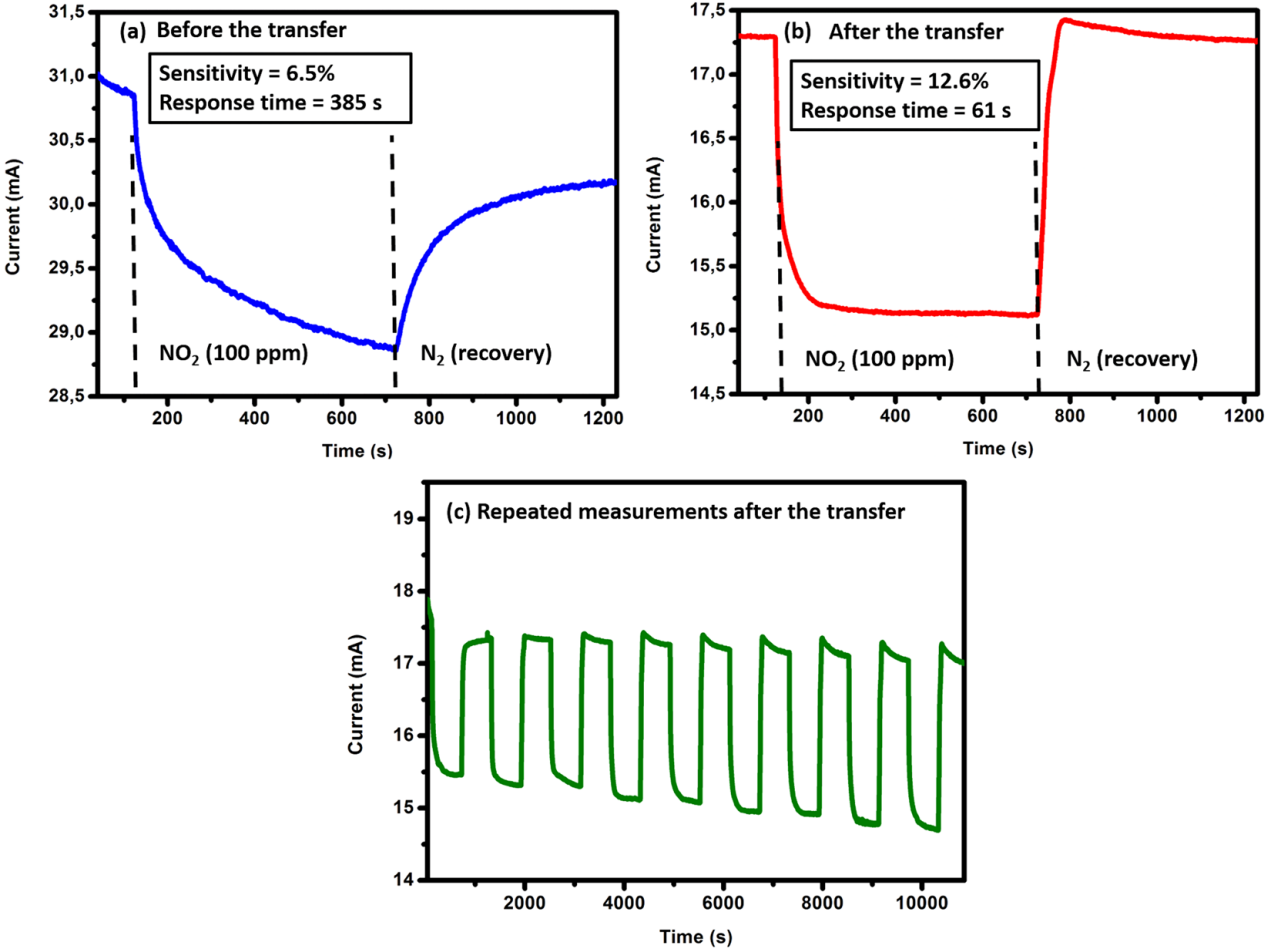
Response of an AlGaN/GaN sensor, with a gate size of 2 μm × 200 μm, to NO_2_ gas for a concentration of 100 ppm at 30 °C. (**a**) Before the transfer, (**b**) after the transfer to the flexible template and (**c**) Repeated measure after the transfer showing the stability of the transferred device after 9 cycles.

AlGaN/GaN gas sensors with greater than 80% gate pinching were tested under NO_2_ gas for a concentration of 100 ppm at 30 °C, both as-grown on the original sapphire substrate and after transfer to a tape with acrylic adhesive. Exposure to the gas leads to decreased drain-source current. From the transient response and recovery curves, we have calculated sensitivity as S = [|I_0_ − I_gas_|**/**I_0_]*100, where I_0_ is the initial current under pure N_2_ and I_gas_ is the steady state current after the test gas has been applied. This metric gives a normalized measure of the change in device current under gas exposure. We have also determined the response time τ defined by the time between 10% and 90% of the initial and final steady state values under gas exposure. The average sensitivity S was found to be 2.8% ± 1.4% with an average response time of 361 s ± 140.8 s for around 20 tested devices. Figure 4a presents one of the best response we obtained before the transfer, S and τ were calculated to be equal to 6.5% and 385 s respectively. Working devices after the transfer to the new substrate have undergone the same testing procedure. The results indicate an average sensitivity S = 12% ± 1.2% with a response time ranging from 7 s to 61 s. Figure 4b shows result from the same device, used in Fig. 4a, after its transfer. This sensor presents a doubling in sensitivity, a six times lower response time and a faster recovery after gas exposure. We also note that a repeated measurement with 9 test cycles during more than three consecutive hours has been performed after the transfer exhibiting similar response and no significant drift, as shown in Fig. 4c.

**Figure 5:**
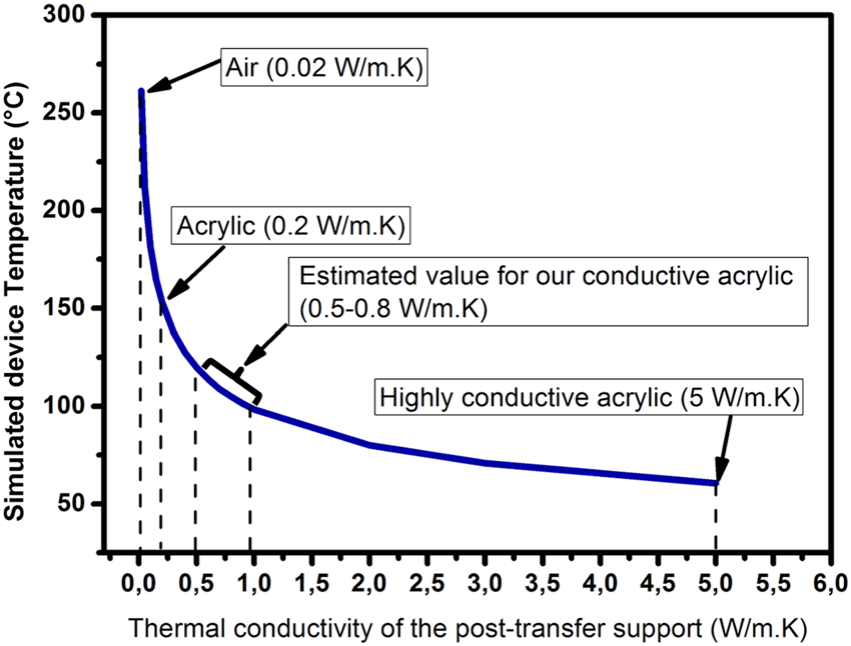
Thermal simulation of the operating device temperature Vs thermal conductivities of different post-transfer supports.

The observed large enhancement in sensitivity and response time after transfer may be attributed to several root causes. Our choice of the final support is expected to enable the confinement of the heat generated by self-heating. As seen in Fig. 5, thermal modeling results show that the tape with electrically conductive acrylic adhesive used in this study is predicted to yield a device operating temperature ranging between 105 °C and 128 °C. In addition, we have performed Raman spectroscopy measurements on the E_2_ GaN line before and after transfer at different temperatures and with different input powers, based on the method reported in ref.^15^. The Raman measurements show that pre-transfer self-heating results in a sensor temperature of 60 °C in the active region. After transfer, the sensor temperature was measured to be 120 °C. These results are consistent with thermal simulations presented in Fig. 5. It also explains the drop in I_0_ after transfer since device temperature is inversely related to the electron mobility in the 2DEG^16^.

To verify whether this increase in device operating temperature is the primary reason behind the boosted performances, we have operated a pre-transfer sensor (on h-BN/sapphire) at 120 °C and compared the performance to the transferred sensor. The sensitivity and the response time of a pre-transfer device were measured as a function of temperature. The results show that the sensitivity has only increased by a factor of 1.06 when varying the stage temperature from 30 °C to 120 °C, and the response time has decreased by a factor of 2.47. Because these factors are less than those resulting from the transfer (1.94 and 6.31, respectively), it suggests that other reasons in addition to thermal effects are behind the post-transfer performance enhancement.

**Figure 6:**
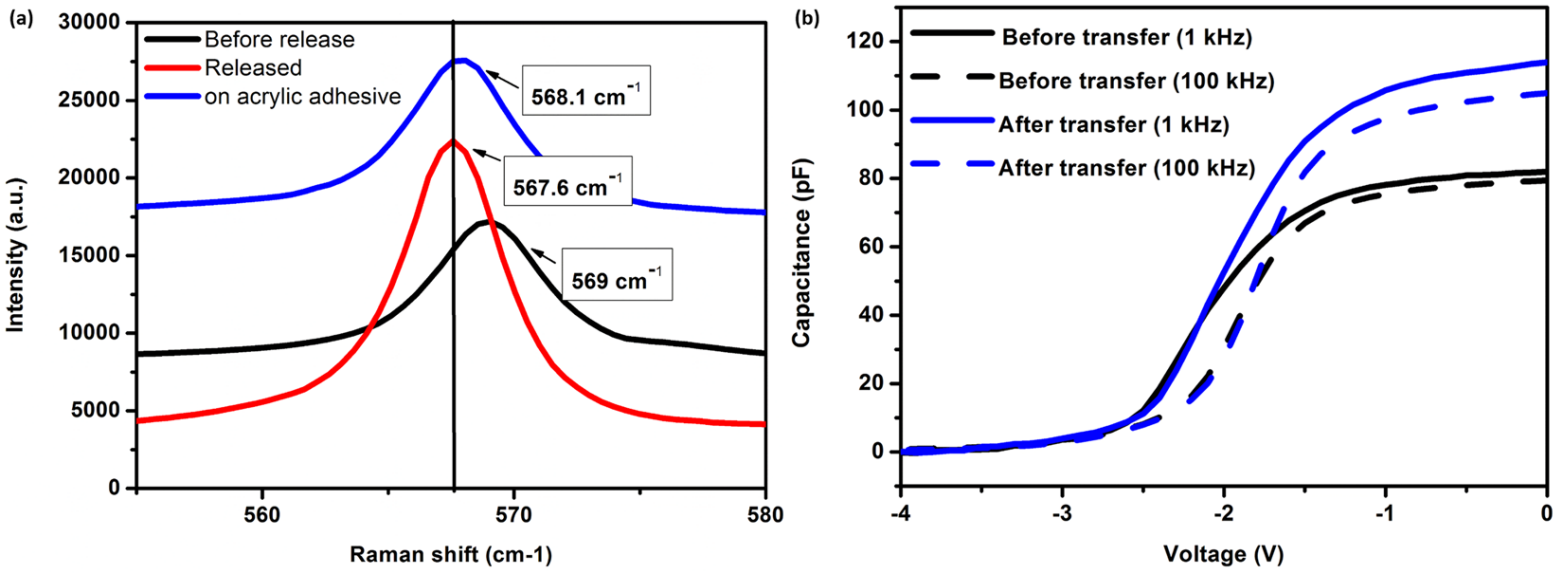
(**a**) Raman spectra at E2 peak of GaN on h-BN/sapphire (black), released from the sapphire (red) and transferred to an adhesive acrylic tape (red). (**b**) Capacitance-voltage measured at 1 kHz and 100 kHz on a device before and after its transfer. Both Raman and C-V results indicate an increase in the 2DEG density after the release and transfer to the acrylic adhesive tape.

The effects from strain changes in the semiconductor layers after the transfer may play a role and should be considered. Raman spectroscopy results, performed at room temperature both before and after transfer, show a relaxation of the GaN layer after the transfer. The E_2_ peak of GaN has shifted towards less compressive strains, as presented in Fig. 6a, which agrees with the reported results in ref.^11^ and indicates an increase of the 2DEG carrier density. This is necessarily linked with an increase in the surface state charges to maintain the electrostatic neutrality^17^, and can consequently contribute to the enhancement in sensitivity since the gas molecules are chemically absorbed to surface charges after dissociation on the Pt sensing layer^18^.

To further confirm the increase in 2DEG carrier density, we have conducted C-V measurements at different frequencies between the gate and the source of a device before and after its transfer to the adhesive tape. From the C-V profiling in Fig. 6b, we observe a 39% increase in maximum capacitance measured at 1 kHz after transfer which is directly related to the 2DEG carrier density at the AlGaN/GaN interface.

It is clear that temperature increase and stress release are both valid causes for the performance boost but other effects as surface charge traps density could be also investigated. Another possible factor for the enhanced sensor performance could be related to modification of the strain in the Pt sensing layer during transfer, which is known to change the catalyst efficiency^19,20^ and hence it may also contribute to the decreased response time. More in depth study should be carried out to better understand the individual contributions of each of these factors to the total increase of device performance.

In conclusion, we have demonstrated wafer-scale fabrication of AlGaN/GaN gas sensors grown on an ultra-thin h-BN layer on a 2-inch sapphire substrate. The h-BN layer allowed the transfer of the devices to a flexible and heat insulating acrylic tape. Gas sensing measurements have been carried out before and after the transfer showing greatly improved performance on the flexible substrate. We have considered the effects of the post-transfer substrate choice on the device operating temperature which has been shown to increase through Raman spectroscopy and thermal simulations. The boosted sensor performance may also be attributed to structural modifications that increase the number or the accessibility of surface states at the platinum/GaN cap interface and/or enhance the catalytic reaction in the sensing layer. The approach for device engineering demonstrated in this work provides new options towards the development of GaN-based flexible sensors with enhanced device performance, improved economic viability, and an expanded range of applications.

## Methods

### Material growth

Materials growth was performed in an Aixtron MOVPE CCS 3 × 2″ system on a 2 inch (0001) sapphire substrate. Triethylboron (TEB), Trimethylgallium (TMGa), Trimethylaluminum (TMAl) and Ammonia (NH_3_), were used as B, Ga, Al and N sources respectively. First, an h-BN layer (3–5 nm) was grown on the sapphire substrate at 1300 °C. Then, a nucleation AlGaN layer (250 nm) with an Al mole fraction of 14% was grown at 1100 °C. We subsequently grew an AlGaN/GaN heterostructure consisting of a 1-µm-thick GaN buffer layer and a 25-nm-thick AlGaN barrier layer with an Al composition of 25%, which was confirmed by XRD measurements. Finally, a 3 nm GaN cap layer was grown. High resolution X-ray diffraction (HRXRD) scans were done in Panalytical X’pert Pro MRD system with Cu Kα radiation.

### Device processing

The use of sacrificial h-BN for wafer-scale fabrication of gas sensors poses a processing challenge because spontaneous delamination of the grown structure can occur during device processing when the wafer is subject to various mechanical strains. In our experience, careful control and optimization of each fabrication step is required. The source, drain, and contacts were defined by optical lithography. The deposit of the source and drain metallization contact structure consisted of electron beam evaporated Ti/Al/Ni/Au (12/200/40/100 nm) multilayers followed by a rapid thermal annealing at 870°C for 30 s under nitrogen atmosphere. The gate contact was deposited using Pt sputtering with a thickness of 15 to 20 nm at a pressure of 6 mTorr, providing a catalytically active sensing layer. Gate dimensions are Lg = 2 µm and Wg = 200 µm, with a total drain to source spacing of 6 µm. Electron beam evaporated Ti/Al/Ni/Au (12/200/40/100 nm) pads were deposited to facilitate electrical contacting of the devices.

After sensor processing, electrical characterization was performed using an automated probe station to test the electrical behavior of the devices. Then, the sapphire layer was removed by fixing supports to both the sapphire and active layer sides with a thermoplastic polymer, and applying pressure so that the Van der Waals bonds in the h-BN layer are broken. The active layers are then transferred to a flexible substrate; we have chosen to use a tape with acrylic adhesive for thermal insulation. The top support is removed by heating the thermoplastic at 60 °C and rinsing the residues with DI water.

### Experimental testing of gas sensors

A subset of the transferred sensor devices has been tested as gas sensors before and after their transfer to copper flexible tape. For experimental testing, the sensors were connected with probes in a gas chamber and linked to a Keithley 236 I-V measurement system. Gas sources of pure N_2_ and NO_2_ with 100 ppm concentration were supplied to the testing chamber via an MCQ gas mixer utilizing separate mass flow controllers for each gas line. Pressure, concentration, temperature, and flow rate were all controlled and kept constant during the measurements. All experiments were carried out at atmospheric pressure. The substrate temperature was controlled using an external temperature controller with a heater and fixed at 30 °C. A flow rate of 100 sccm was used and with all external factors controlled, we can attribute changes in the steady state signal to the gas detection mechanism described in [2]. For each measurement, the signal under pure N_2_ was used as a reference for comparison with the signal under the test gas in a background of nitrogen.

### Thermal modeling

Thermal simulations have been performed along with the temperature measurements made on pre- and post-transfer devices by means of Raman spectroscopy. One would expect that the primary difference between the two devices would be a higher temperature post-transfer, because of the low thermal conductivity of the 35 micron thick electrically conductive acrylic adhesive between the transferred devices and the copper film. The simulations show that this is the case. In addition, they show that in the vertical direction in the AlGaN/GaN structure, the temperature is very nearly uniform, with less than 0.1 °C temperature difference, which implies that the vertically averaged temperature measured by Raman spectroscopy is within 0.1 °C of that of the Pt/AlGaN interface. Thermal conductivities used in the simulation are 130 W/m.K for GaN, 25 W/m.K for Al_0.14_Ga_0.86_N, 20 W/m.K for Al_0.25_Ga_0.75_N, 19 W/m.K for Ti, 205 W/m.K for Al, 90 W/m.K for Ni, 314 W/m.K for Au, 72 W/m.K for Pt, and 385 W/m.K for copper. The temperature at the 25-micron thick copper film is 30 °C, the same as the chuck holder.

In simulations, the temperature at the Pt/AlGaN interface on a post-transfer device during 5 V operation depends strongly on the thermal conductivity of the electrically conductive acrylic adhesive. Non-conductive acrylic has a thermal conductivity of approximately 0.2 W/m.K, while the thermal conductivity of commercial conductive acrylic adhesives ranges from 0.5 to 5 W/m.K. As our tape is not meant to be highly thermally conductive, its thermal conductivity is estimated to be between 0.5 and 0.8 W/m.K which is consistent with Raman results.

### Raman spectroscopy

Raman shift spectra were measured by LabRam HR EVOLUTION Raman spectroscopy with laser excitation at 532 nm. For calibration, the GaN E_2_ Raman shift as a function of temperature was measured through the platinum gate on a non-operating sensor before lift-off. The E_2_ shift moved from 568.25 cm^−1^ at 30 C to 564 cm^−1^ at 300 C in a line. Then with the sample holder set to 30 C, the Raman shift was measured during operation with V_DS_ = 5 V, before and after the transfer.
